# Supplementary material for: Genome-Wide Identification and Characterization of Homeobox Transcription Factors in Phoma sorghina var. saccharum Causing Sugarcane Twisted Leaf Disease
Source: Int J Mol Sci. 2024 May 14;25(10):5346. doi: 10.3390/ijms25105346 (PMC11121360; doi:10.3390/ijms25105346)
Supplement: Supplementary file 1 [file ijms-25-05346-s001.zip › ijms-2978935-supplementary.pdf]

Supplementary Figures:

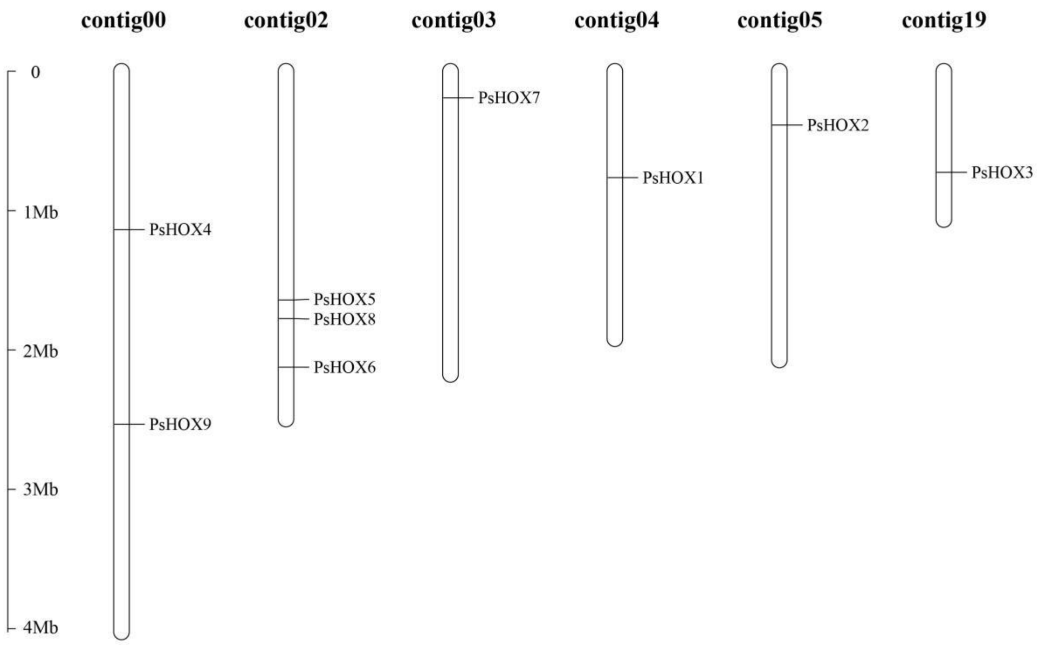

Figure S1. Chromosomal locations of *HOX* genes are shown in *Phoma sorghina* var. *saccharum* BS2-1 genome.

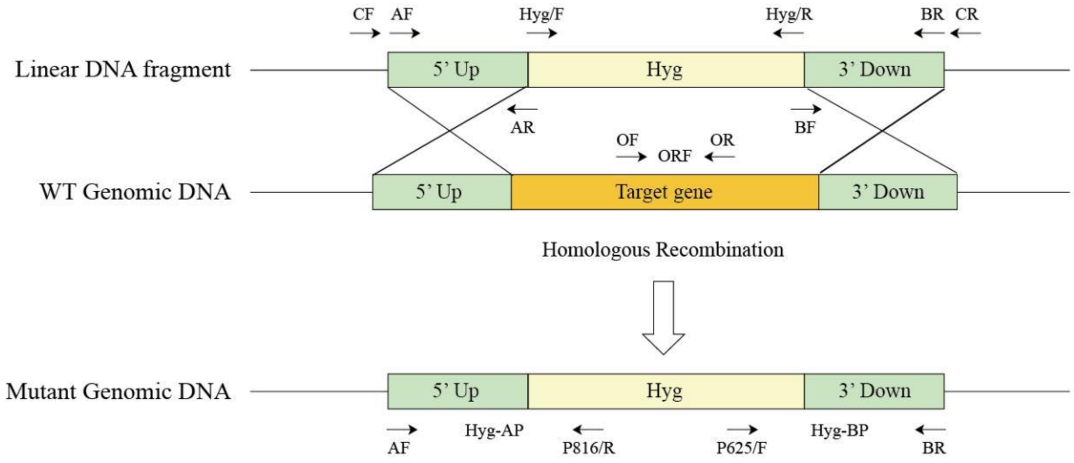

Figure S2. The gene replacement strategy of *HOX*.

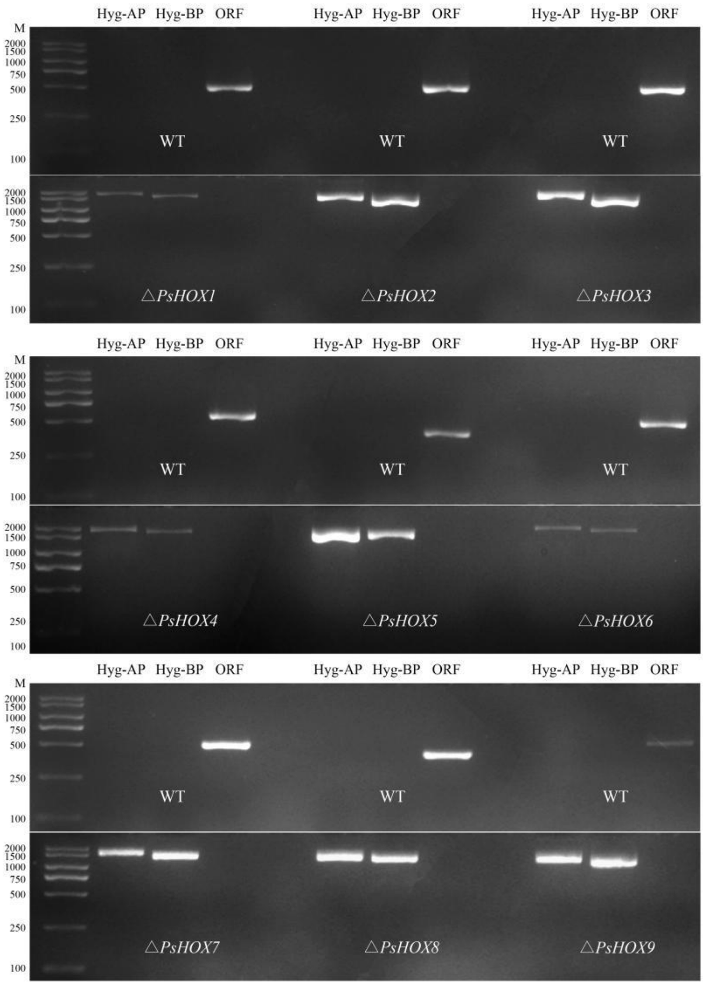

Figure S3. Confirmation of HOX genes deletion in BS2-1 using diagnosed PCR. Hyg-AP, Hyg-BP, and ORF, represent the indicated DNA fragments shown in Figure S5.

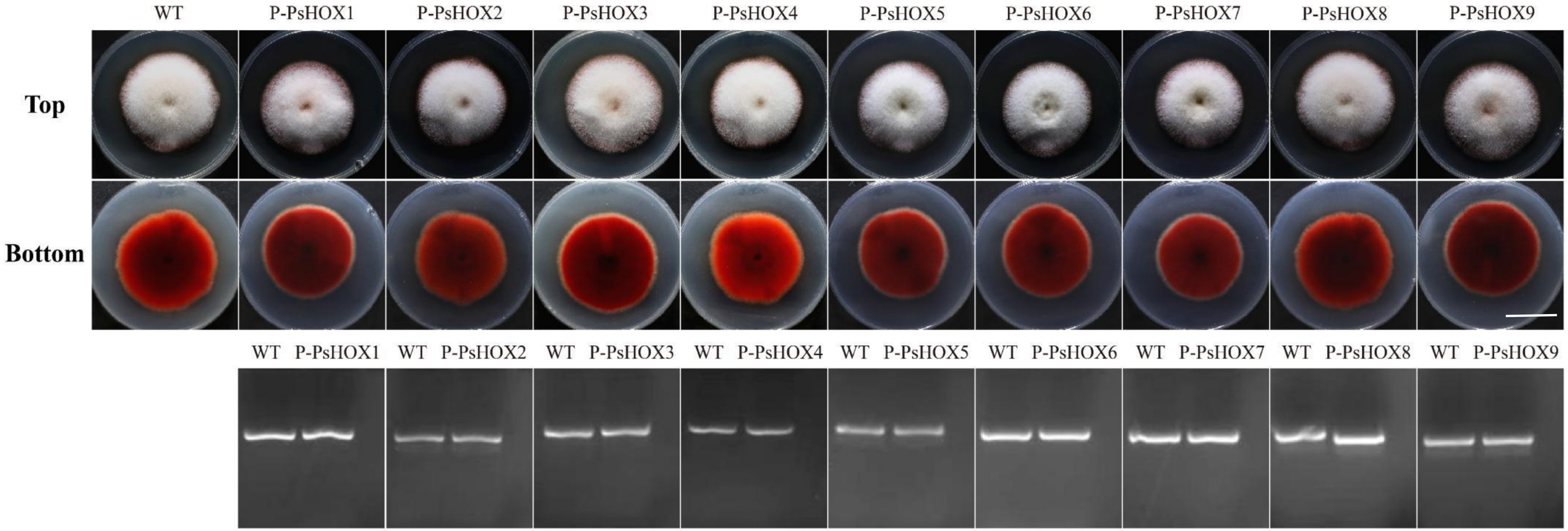

Figure S4. Confirmation of *HOX* genes complement in *Phoma sorghina* var. *saccharum* BS2-1 using phenotype observed (up) and diagnosed PCR (down). Bars=3 cm.

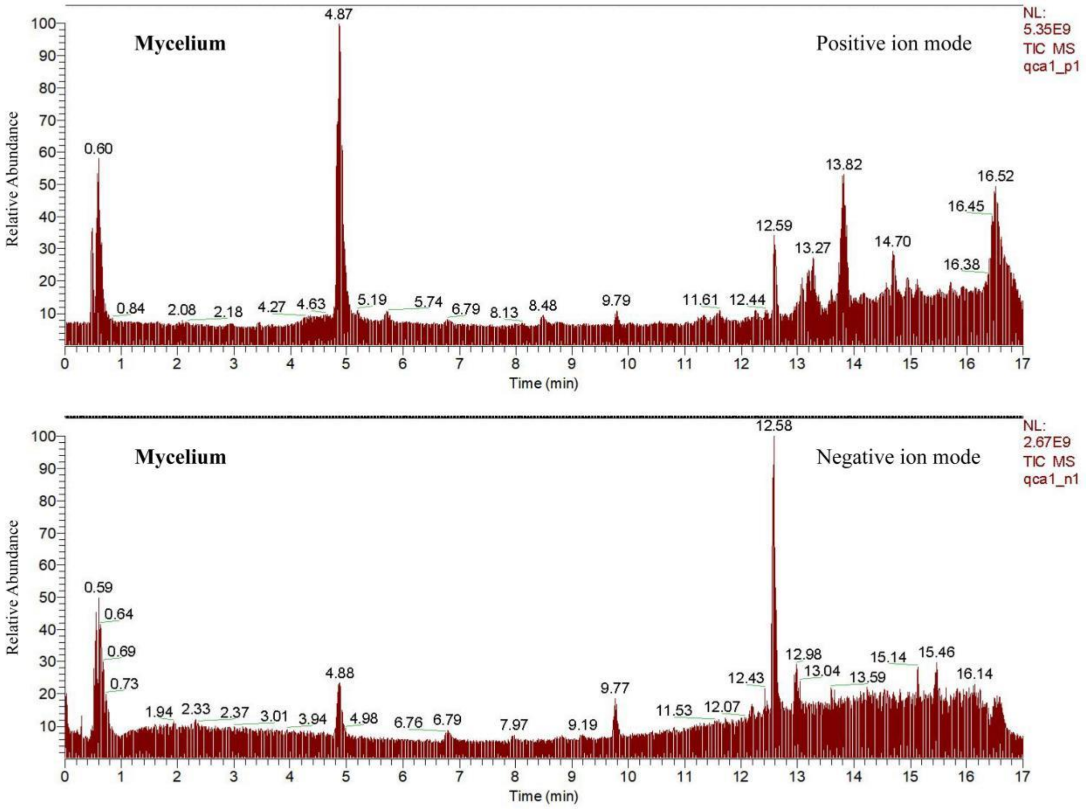

Figure S5. Total ion chromatogram (TIC) of *P. sorghina* BS2-1 and four mutants (*ΔPsHOX1*, *ΔPsHOX3*, *ΔPsHOX6*, *ΔPsHOX9*) from mycelium.

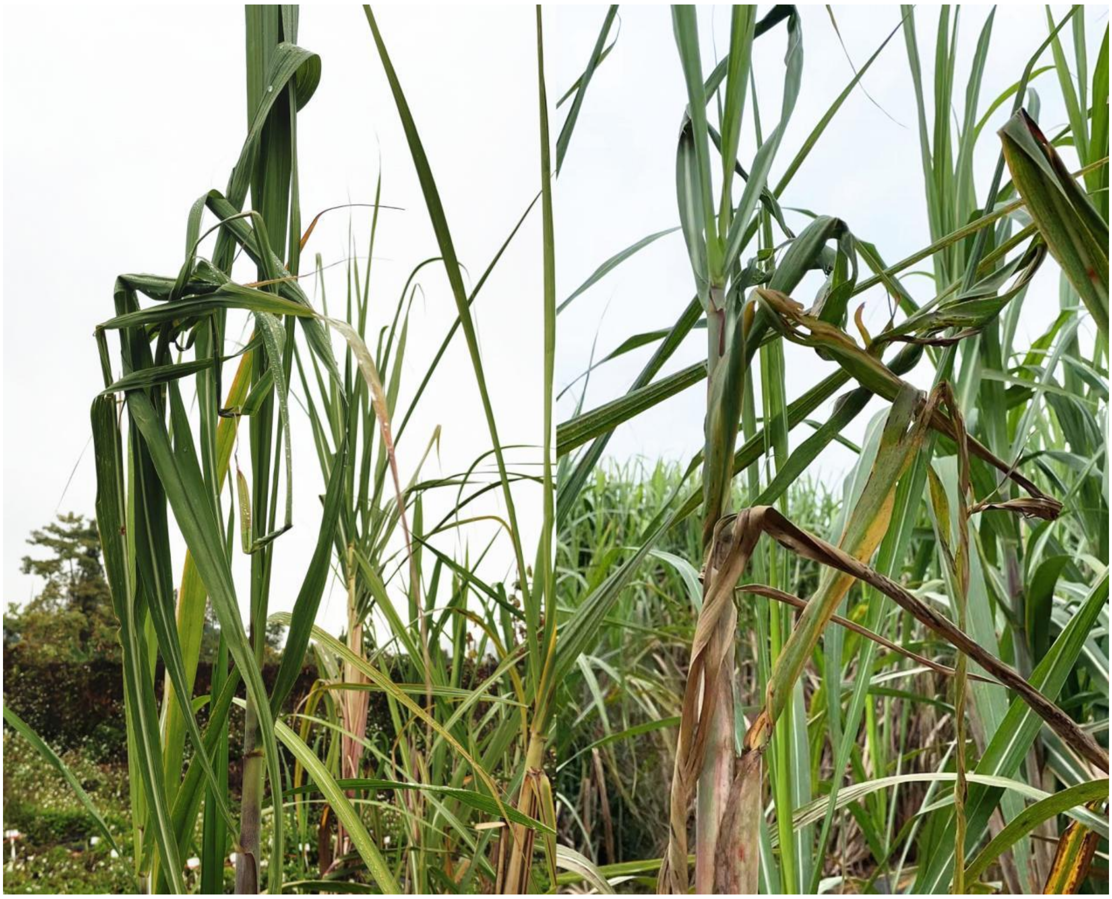

Figure S6. Typical twisting and curling of crown leaves symptoms in field sugarcane.

Supplementary Tables:

Table S1. Global statistics for the *P. sorghina* BS2-1 genome assembly, BUSCO analysis and the length of chromosomes.

	telomeric repeat 3′	telomeric repeat 5′	Length (bp)
Contig 00	+	+	4,023,921
Contig 01	+	+	2,588,534
Contig 02	+	−	2,497,106
Contig 03	+	−	2,176,165
Contig 04	+	−	1,920,476
Contig 05	+	−	2,072,022
Contig 07	+	−	1,973,798
Contig 08	+	+	1,858,818
Contig 10	+	+	1,705,504
Contig 13	+	+	1,602,205
Contig 14	+	−	1,419,212
Contig 16	+	+	1,386,965
Contig 17	+	+	1,316,475
Contig 18	+	−	693,251
Contig 19	+	+	1,064,691
Contig 20	+	−	473,809
Contig 21	+	+	1,085,172
Contig 22	+	−	1,084,869
Contig 23	+	+	1,108,709
Contig 25	+	+	977,488
Contig 29	−	−	28,739
Mitochondrion			66,703
Total			33,124,632
**Summary**			
Genome size (bp)			33,124,632
Depth of coverage			97 ×
Contig number			22
Contig N50 (bp)			1,920,476
Contig N90 (bp)			1,084,869
Contig max (bp)			4,023,921
GC content			52.12 %
Complete BUSCOs (C)			286 (98.6 %)
Complete and single-copy BUSCOs (S)			286 (98.6 %)
Complete and duplicated BUSCOs (D)			0
Fragmented BUSCOs (F)			1 (0.3 %)
Missing BUSCOs (M)			3 (1.1 %)
Total BUSCO groups searched			290

Note: + shows that chromosomes with telomeric repeats sequences (TTAGGG) on the ends; −shows that chromosomes can not found the complete telomeric repeats sequences (TTAGGG) on the ends.

Table S2. Functional annotation of predicted protein-coding genes in *P. sorghina* BS2-1.

Method	Software		P. sorghina BS2-1
Ab initio	Augustus	-	13,349
	Glimmer HMM	-	7,788
	SNAP	-	10,808
Homolog-based	GeMoMa	Ascochyta rabiei	9,952
Integration	EVM	-	7,973
Gene length (bp)			17,689,450
Average gene length (bp)			2,219
Exon length (bp)			13,034,658
Average exon length (bp)			595.3
Intron length (bp)			21,895
Average Intron length (bp)			212.6
**Database**			
GO (Gene Ontology)			4,415 (55.4 %)
COG (Cluster of Orthologous Groups of proteins)			3,556 (44.6 %)
KEGG (Kyoto Encyclopedia of Genes and Genomes)			2,767 (34.7 %)
Pfam			6,169 (77.4 %)
Swiss-Prot			5,239 (65.7 %)
TrEMBL			7,854 (98.5 %)
Nr (non-redundant proteins)			7,851 (98.5 %)
CAZyme (Carbohydrate-Active EnZymes Database)			515 (6.5 %)
PHI (Pathogen–Host Interactions Database)			2,440 (30.6 %)
TCDB (Transporter Classification Database)			115 (1.4 %)
SP (Secreted protein)			583 (7.3 %)
tRNA			168 (2.1 %)
rRNA			113 (1.4 %)
Pseudogene			76 (1.0 %)

Table S3. Repeat sequences identification and classification in *P. sorghina* BS2-1 genome.

Type	Number	Length (bp)	Rate (%)
ClassI/?/?	25	30,265	0.09
ClassI/LINE/I	8	731	0
ClassI/LINE/Jockey	1	57	0
ClassI/LINE/L1	7	490	0
ClassI/LTR/?	7	1,066	0
ClassI/LTR/Copia	34	4,968	0.01
ClassI/LTR/Gypsy	158	107,912	0.33
ClassI/PLE/Penelope	32	4,302	0.01
ClassI/SINE/?	50	3,266	0.01
ClassII/?/?	1	170	0
ClassII/?/Kolobok	3	120	0
ClassII/Helitron/Helitron	3	199	0
ClassII/TIR/CACTA	4	151	0
ClassII/TIR/P	9	511	0
ClassII/TIR/PIF-Harbinger	2	140	0
ClassII/TIR/Tc1-Mariner	57	52,395	0.16
ClassII/TIR/hAT	10	835	0
**Total**	**411**	**207,578**	**0.63**

Table S4. The statistical results of transcription factors identified in *P. sorghina* BS2-1.

**Families**	**Number**	**Families**	**Number**
AF-4	3	Others	3
ARID	2	RFX	1
bHLH	8	SRF	2
Fork head	3	TEA	1
HMG	9	TSC22	1
HMGA	1	ZBTB	15
Homeobox	9	zf-BED	1
HSF	3	zf-C2H2	39
MYB	19	zf-CCCH	8
NDT80_PhoG	1	zf-GAGA	1
NF-YA	1	zf-GATA	6
NF-YB	3	zf-LITAF-like	1
NF-YC	3	zf-MIZ	3
**Total**			**147**

Table S5. The Ct value of wild-type strain and mutants to sugarcane.

Sample	Ct value*	Status	Sample	Ct value*	Status
WT_D1	17.94	Infected	△*PsHOX5*_D1	17.54	Infected
WT_D2	19.45	Infected	△*PsHOX5*_D2	19.09	Infected
WT_D3	17.34	Infected	**△*PsHOX5*_D3**	**35.21**	**Healthy**
WT_D4	18.99	Infected	**△*PsHOX5*_D4**	**36.94**	**Healthy**
WT_D5	17.22	Infected	△*PsHOX5*_D5	18.98	Infected
WT_D6	17.86	Infected	△*PsHOX5*_D6	19.35	Infected
WT_D7	18.09	Infected	△*PsHOX5*_D7	20.34	Infected
**WT_D8**	**36.51**	**Healthy**	△*PsHOX5*_D8	21.87	Infected
WT_D9	18.42	Infected	△*PsHOX5*_D9	20.88	Infected
WT_D10	17.02	Infected	△*PsHOX5*_D10	23.98	Infected
△*PsHOX1*_D1	25.32	Infected	**△*PsHOX6*_D1**	**35.65**	**Healthy**
**△*PsHOX1*_D2**	**37.22**	**Healthy**	**△*PsHOX6*_D2**	**36.22**	**Healthy**
△*PsHOX1*_D3	26.43	Infected	**△*PsHOX6*_D3**	**35.55**	**Healthy**
△*PsHOX1*_D4	25.33	Infected	**△*PsHOX6*_D4**	**35.71**	**Healthy**
**△*PsHOX1*_D5**	**37.53**	**Healthy**	**△*PsHOX6*_D5**	**36.20**	**Healthy**
**△*PsHOX1*_D6**	**37.98**	**Healthy**	**△*PsHOX6*_D6**	**36.09**	**Healthy**
△*PsHOX1*_D7	20.23	Infected	△*PsHOX6*_D7	25.09	Infected
△*PsHOX1*_D8	23.31	Infected	**△*PsHOX6*_D8**	**36.21**	**Healthy**
△*PsHOX1*_D9	20.15	Infected	**△*PsHOX6*_D9**	**35.15**	**Healthy**
△*PsHOX1*_D10	19.88	Infected	**△*PsHOX6*_D10**	**36.92**	**Healthy**
△*PsHOX2*_D1	20.43	Infected	△*PsHOX7*_D1	21.65	Infected
△*PsHOX2*_D2	19.22	Infected	△*PsHOX7*_D2	19.98	Infected
△*PsHOX2*_D3	26.65	Infected	△*PsHOX7*_D3	20.54	Infected
△*PsHOX2*_D4	24.55	Infected	△*PsHOX7*_D4	20.17	Infected
**△*PsHOX2*_D5**	**35.64**	**Healthy**	**△*PsHOX7*_D5**	**35.97**	**Healthy**
**△*PsHOX2*_D6**	**36.21**	**Healthy**	△*PsHOX7*_D6	23.53	Infected
△*PsHOX2*_D7	23.43	Infected	**△*PsHOX7*_D7**	**36.01**	**Healthy**
**△*PsHOX2*_D8**	**35.90**	**Healthy**	△*PsHOX7*_D8	20.44	Infected
△*PsHOX2*_D9	20.11	Infected	△*PsHOX7*_D9	21.32	Infected
△*PsHOX2*_D10	19.32	Infected	△*PsHOX7*_D10	19.68	Infected
**△*PsHOX3*_D1**	**35.43**	**Healthy**	**△*PsHOX8*_D1**	**35.98**	**Healthy**
**△*PsHOX3*_D2**	**36.01**	**Healthy**	**△*PsHOX8*_D2**	**36.02**	**Healthy**
**△*PsHOX3*_D3**	**36.99**	**Healthy**	△*PsHOX8*_D3	21.89	Infected
**△*PsHOX3*_D4**	**36.29**	**Healthy**	△*PsHOX8*_D4	20.47	Infected
**△*PsHOX3*_D5**	**35.56**	**Healthy**	△*PsHOX8*_D5	21.98	Infected
△*PsHOX3*_D6	27.91	Infected	△*PsHOX8*_D6	19.55	Infected
△*PsHOX3*_D7	28.99	Infected	△*PsHOX8*_D7	22.45	Infected
**△*PsHOX3*_D8**	**36.43**	**Healthy**	**△*PsHOX8*_D8**	**35.99**	**Healthy**
**△*PsHOX3*_D9**	**35.55**	**Healthy**	△*PsHOX8*_D9	23.01	Infected
**△*PsHOX3*_D10**	**35.98**	**Healthy**	△*PsHOX8*_D10	22.95	Infected
△*PsHOX4*_D1	26.54	Infected	**△*PsHOX9*_D1**	**35.99**	**Healthy**
△*PsHOX4*_D2	23.43	Infected	**△*PsHOX9*_D2**	**35.32**	**Healthy**

△*PsHOX4*_D3	27.09	Infected	**△*PsHOX9*_D3**	**37.19**	**Healthy**
△*PsHOX4*_D4	28.33	Infected	**△*PsHOX9*_D4**	**36.96**	**Healthy**
**△*PsHOX4*_D5**	**35.06**	**Healthy**	**△*PsHOX9*_D5**	**36.01**	**Healthy**
**△*PsHOX4*_D6**	**36.37**	**Healthy**	△*PsHOX9*_D6	24.15	Infected
△*PsHOX4*_D7	23.98	Infected	**△*PsHOX9*_D7**	**35.82**	**Healthy**
△*PsHOX4*_D8	24.09	Infected	**△*PsHOX9*_D8**	**36.98**	**Healthy**
**△*PsHOX4*_D9**	**37.01**	**Healthy**	**△*PsHOX9*_D9**	**35.31**	**Healthy**
△*PsHOX4*_D10	26.94	Infected	**△*PsHOX9*_D10**	**36.79**	**Healthy**
**CK1**	**37.02**	**Healthy**	**CK6**	**35.64**	**Healthy**
**CK2**	**36.22**	**Healthy**	**CK7**	**36.90**	**Healthy**
**CK3**	**35.79**	**Healthy**	**CK8**	**36.43**	**Healthy**
**CK4**	**37.98**	**Healthy**	**CK9**	**36.32**	**Healthy**
**CK5**	**36.11**	**Healthy**	**CK10**	**35.97**	**Healthy**

* Ct value < 35 (infected)

Table S6. The pathogenicity of wild-type strain and mutants to sugarcane.

Strains	Incidence rate (%)
WT (BS2-1)	90.00
△*PsHOX1*	70.00
△*PsHOX2*	70.00
△*PsHOX3*	20.00
△*PsHOX4*	70.00
△*PsHOX5*	80.00
△*PsHOX6*	10.00
△*PsHOX7*	80.00
△*PsHOX8*	70.00
△*PsHOX9*	10.00
CK	0.00

Table S7. Gene-specific primers for gene knock-out.

Primer name	Sequence (5′-3′)	Target gene	Size (bp)
PHOM_00410/AF	CGATCACAGCTAGCCAGCTG	*PsHOX1* A fragment	1,000
PHOM_00410/AR	TCTTTCTAGAGGATCCCCGGGTACATCCCATGTTCCTCAGGCAAGC		
PHOM_00410/BF	ATATCATCTTCTGTCGACCTGCAGGCGTCGCCGCCAGGCTTC	*PsHOX1* B fragment	1,000
PHOM_00410/BR	GAACAGCAGCAACGACGACAACTG		
PHOM_00410/CF	CGATCACAGCTAGCCAGCTG	*PsHOX1* A-target gene-PsHOX1-B gene cassette	4,400
PHOM_00410/CR	GAACAGCAGCAACGACGACA		
PHOM_00410/OF	ATTGGCTCTGACTGAACCAGGC	*PsHOX1* ORF	544
PHOM_00410/OR	GCAGGACTTGACACGGAAACAC		
PHOM_01626/AF	GCTGGATGCAAATGAGAACAGGA	*PsHOX2* A fragment	1,000
PHOM_01626AR	TCTTTCTAGAGGATCCCCGGGTACTCATGTTCGGGTAGAGTAAGGA		
PHOM_01626/BF	ATATCATCTTCTGTCGACCTGCAGGGTTATTCGCGACTGTATCTGGCA	*PsHOX2* B fragment	1,000
PHOM_01626/BR	CAGTTGGAAGAGAGCAAAAGGA		
PHOM_01626/CF	GCTGGATGCAAATGAGAACAGGA	*PsHOX2* A-target gene-PsHOX2-B gene cassette	3,809
PHOM_01626/CR	CAGTTGGAAGAGAGCAAAAGGA		
PHOM_01626/OF	TGTCGTGCCTGATTCTGCGC	*PsHOX2* ORF	553
PHOM_01626/OR	CGCGAACCTCATGGATCTCC		
PHOM_02470/AF	CCTGAAGCAGACCTGGCCG	PsHOX3 A fragment	1,000
PHOM_02470/AR	TCTTTCTAGAGGATCCCCGGGTACGTTGGGGAGGTGGACACCTG		
PHOM_02470/BF	ATATCATCTTCTGTCGACCTGCAGGTGGCATTCTGGTTTCGCGTT	*PsHOX3* B fragment	1,000
PHOM_02470/BR	GCGCGGAAAACAAACTAGAAAGT		
PHOM_02470/CF	CCTGAAGCAGACCTGGCCG	*PsHOX3* A-target gene-PsHOX3-B gene cassette	3,857
PHOM_02470/CR	GCGCGGAAAACAAACTAGAAAGT		
PHOM_02470/OF	TCAACTTCCGCCCTGCCTCA	*PsHOX3* ORF	479
PHOM_02470/OR	TGCCGAGTGCGGAGGAG		
PHOM_03333/AF	CCTTCAGGGACCAGTGAGAGG	*PsHOX4* A fragment	1,000
PHOM_03333/AR	TCTTTCTAGAGGATCCCCGGGTACTCTGTGAGAGTGTGCGGGAT		
PHOM_03333/BF	ATATCATCTTCTGTCGACCTGCAGGTTTTGTCTCTGGTTGGACCAAATTTGT	*PsHOX4* B fragment	1,000
PHOM_03333/BR	CCCTGGCACATTCGGTTTCG		
PHOM_03333/CF	CCTTCAGGGACCAGTGAGAGG	*PsHOX4* A-target gene-PsHOX4-B gene cassette	3,454
PHOM_03333/CR	CCCTGGCACATTCGGTTTCG		
PHOM_03333/OF	TTCCTTTGCTCAGCCACCCC	*PsHOX4* ORF	366
PHOM_03333/OR	ACCTGGTTCAATGTCAAGTTTGTCAG		

PHOM_04409/AF	ATCGAAGGCTACTGGAACGGC	*PsHOX5* A fragment	1,000
PHOM_04409/AR	TCTTTCTAGAGGATCCCCGGGTACTTATCTCTCTTTTCCGGCGTTCG		
PHOM_04409/BF	ATATCATCTTCTGTCGACCTGCAGGGTGCTCGGCGCTCGACAA	*PsHOX5* B fragment	1,000
PHOM_04409/BR	AGCTACTTCGTGGTTCTCACTTGC		
PHOM_04409/CF	ATCGAAGGCTACTGGAACGGC	*PsHOX5* A-target gene-PsHOX5-B gene cassette	3,611
PHOM_04409/CR	AGCTACTTCGTGGTTCTCACTTGC		
PHOM_04409/OF	AGAGTGGTTGTTCGCACTTATGAT	*PsHOX5* ORF	531
PHOM_04409/OR	TGACCACATGGTTCACCAACATTCG		
PHOM_05072/AF	ACAGGCGCACAGTGCGG	*PsHOX6* A fragment	1,000
PHOM_05072/AR	TCTTTCTAGAGGATCCCCGGGTACGCTGGCGAACGGCCCCTT		
PHOM_05072/BF	ATATCATCTTCTGTCGACCTGCAGGAGCTACCTCTTGCAGGCCAC	*PsHOX6* B fragment	1,000
PHOM_05072/BR	AAGAGATGATATGCGTAGATGGTCCT		
PHOM_05072/CF	ACAGGCGCACAGTGCGG	*PsHOX6* A-target gene-PsHOX6-B gene cassette	4,686
PHOM_05072/CR	AAGAGATGATATGCGTAGATGGTCCTATTCTCGT		
PHOM_05072/OF	AGATGCATCCCGACGACG	*PsHOX6* ORF	508
PHOM_05072/OR	CGGCAACTCCATACACGGC		
PHOM_05197/AF	ATCTCGGGGCCACAAAAGAA	*PsHOX7* A fragment	1,000
PHOM_05197/AR	TCTTTCTAGAGGATCCCCGGGTACTGTAGTGTTTCATAGACACAGACAAGTT		
PHOM_05197/BF	ATATCATCTTCTGTCGACCTGCAGGCCTCTAATGATGCAAGCAGACTGT	*PsHOX7* B fragment	1,000
PHOM_05197/BR	CAGCTGCCTTCCAGAGCTCG		
PHOM_05197/CF	ATCTCGGGGCCACAAAAGAAG	*PsHOX7* A-target gene-PsHOX7-B gene cassette	5,701
PHOM_05197/CR	CAGCTGCCTTCCAGAGCTCG		
PHOM_05197/OF	TCTGCAATGATTCTCTTCCTCTCCTCC	*PsHOX7* ORF	527
PHOM_05197/OR	CGACTTCGTCGTGAAGATCCAATTGA		
PHOM_06420/AF	ATTCGGCCTATGATCGTCGGT	*PsHOX8* A fragment	1,000
PHOM_06420/AR	TCTTTCTAGAGGATCCCCGGGTACGTGGGGTCTACGAAGAATGCA		
PHOM_06420/BF	ATATCATCTTCTGTCGACCTGCAGGCGTGGTAGCCTTTCGGGACT	*PsHOX8* B fragment	1,000
PHOM_06420/BR	AGTCGATATGTTCAGTTCAGTGCG		
PHOM_06420/CF	ATTCGGCCTATGATCGTCGGT	*PsHOX8* A-target gene-PsHOX8-B gene cassette	5,092
PHOM_06420/CR	AGTCGATATGTTCAGTTCAGTGCG		
PHOM_06420/OF	TACTAGGTTCAAGTTCGCCGCAATAC	*PsHOX8* ORF	492
PHOM_06420/OR	CTCTTACAGCTATTGCCCAGGCC		
PHOM_07085/AF	GACACGTTTGAGGGAGAGCCA	*PsHOX9* A fragment	1,000
PHOM_07085/AR	TCTTTCTAGAGGATCCCCGGGTACGAGGGAGGGGGGTTCTGGC		

PHOM_07085/BF	ATATCATCTTCTGTCGACCTGCAGGCTCATTAAACCGATTGTATGCGTGAAA	*PsHOX9* B fragment	1,000
PHOM_07085/BR	CCTAGCTTGGTCGCAAGTACT		
PHOM_07085/CF	GACACGTTTGAGGGAGAGCCA	*PsHOX9* A-target gene-PsHOX9-B gene cassette	6,059
PHOM_07085/CR	CCTAGCTTGGTCGCAAGTACT		
PHOM_07085/OF	ACGAGCCAGCACGTTCTTTGA	*PsHOX9* ORF	424
PHOM_07085/OR	AGCATGCAACTCGAAGCTTGTC		
Hyg/F	CGGTACCCGGGGATCCTCTA	Hygromycin gene	2,145
Hyg/R	GCCTGCAGGTCGACAGAAGA		
P816/R	ATGGCTGTGTAGAAGTACTCGCC	Hyg-AP fragment (amplified with Hyg/AF)	A+816
P625/F	CTGAATTCCCCAATGTCAAGCACT	Hyg-BP fragment (amplified with Hyg/BR)	B+625
